# Physical Activity and Health-Related Quality of Life Among Low-Income Adults in Metropolitan Kuala Lumpur

**DOI:** 10.2188/jea.JE20170183

**Published:** 2019-02-05

**Authors:** Tin Tin Su, Meram Azzani, Adeoye Philip Adewale, Nithiah Thangiah, Rosilawati Zainol, Hazreen Majid

**Affiliations:** 1 Centre for Population Health (CePH), Department of Social and Preventive Medicine, Faculty of Medicine, University of Malaya, Kuala Lumpur, Malaysia; 2 Department of Nutrition, Harvard Chan School of Public Health, Boston, MA, USA; 3 Centre for Sustainable Urban Planning, Faculty of Built Environment, University of Malaya, Kuala Lumpur, Malaysia; 4 Department of Urban and Regional Planning, Faculty of Built Environment, University of Malaya, Kuala Lumpur, Malaysia; 5 Community Medicine Department, Faculty of Medicine, MAHSA University, Selangor State, Malaysia

**Keywords:** physical activity, quality of life, low income, mental health, Malaysia

## Abstract

**Background:**

The aim of this research is to assess the level of physical activity (PA) in relation to different socio-economic factors and to examine the effect of the recommended level of PA on the domains of quality of life (QoL) among residents of low-income housing in the metropolitan area of Kuala Lumpur, Malaysia.

**Methods:**

This was a cross-sectional study that included 680 respondents from community housing projects. Reported PA was assessed using the Global Physical Activity Questionnaire (GPAQ) short form version 2. The SF-12v2 was administered to assess the health-related QoL (HRQoL) among the study population. Respondents were grouped into “active” and “insufficient” groups according to reported weekly PA level. One-way analysis of variance, analysis of co-variance, and multiple linear regression were used in the analysis.

**Results:**

Overall, 17.6% (95% CI, 14.3–20.9) of the respondents did not achieve the recommended levels of PA (≥600 metabolic equivalent [MET]-minutes week^−1^). Level of achieving recommended PA was higher among younger participants, females, members belonging to nuclear families, and in self-employed participants. The group that fulfilled recommended PA levels (active) has higher levels of QoL in all domains except physical functioning.

**Conclusions:**

Almost one out of five low-income urban residents were physically inactive. In addition, individuals who attained recommended PA levels had better scores on some domains of HRQOL than those who did not. Our findings call for tailor-made public health interventions to improve PA levels among the general population and particularly for low-income residents.

## INTRODUCTION

The Gerontological Society of America, whose motto is “adding life to years, not just more years to life”, should not only be made the guiding principle for the elderly but also the cardinal truth from cradle to grave.^1^ The experiences or choices of living style of individuals and communities significantly contribute to healthy life, since the World Health Organization (WHO) definition of health is “a state of complete physical, mental and social well-being and not merely the absence of disease or infirmity”.^2^ Thus, the crude assessments of health, such as morbidity and mortality, are gradually being replaced with quality of life (QoL) as a more desirable measure of healthy living.

Physical activity (PA) has been said to reduce depression, improves cognitive function, mood, self-esteem, general mental health, short- and long-term memory, sleep, and a general feel of a disease-free life.^3^ It decreases the risk of cardiovascular diseases, hypertension, diabetes mellitus, and lung and bone diseases.^4^ It also reduces cancer all-cause mortality and risk of colon and breast cancers.^5^

Malaysia is an upper-middle income country undergoing rapid demographic, economic, and social changes.^6^ These changes have led to rapid urbanization and poorer lifestyle choices, such as unhealthy dietary habits and inadequate PA.^7^

Nationwide surveys have highlighted an increasing trend of disease burden and prevalence among the Malaysian population.^8^ The Fourth National Health and Morbidity Survey of Malaysia reported that the prevalence of non-communicable disease (NCD) risk factors of obesity, hypercholesterolemia, and hypertension were 27.2%, 35.1%, and 32.7%, respectively, in Malaysia.^9^ The respondents from the urban areas reported higher prevalence of chronic illness compared to those from rural areas.^8^

A study among pre-diabetics in a semi-urban community in Malaysia indicated that about 60.8% were physically inactive, with a mean PA of <600 metabolic equivalent (MET)-minutes/week.^10^ Another recent study conducted within the Klang Valley area had indicated that one out of five low-income urban dwellers has a high chance of having cardiovascular disease within 10 years.^11^ In addition, some shocking findings from a study among low-income communities in urban Kuala Lumpur reported the prevalence of obesity, hypercholesterolemia, hypertension, and diabetes to be 54.8%, 51.5%, 39.3%, and 7.8%, respectively.^12^ These survey findings underline the need to specifically improve efforts to prevent and control the burden of NCD among low-income communities in urban Malaysia.

The modification of lifestyle could be a cost-effective method to improve health and QoL.^3^ Many people at high risk of chronic diseases exhibit sedentary behavior.^3^ In Malaysia, there is also an increasing trend of mental health problems among adults, from 10.7% in 1996 to 29.2% in 2015.^13^ The prevalence is higher among adults from low-income families and among younger age groups.^13^ Exercise was shown to reduce mental health problems, such as anxiety and depression.^14^ However, PA levels among low-income urban adults are largely unknown; determining such levels is necessary for the development of evidence-based public health intervention for this vulnerable community. Thus, the aim of this study is to assess the reported PA in relation to different socio-economic factors and to examine the effect of the recommended level of PA on the domains of HRQoL among residents of low-income housing in the metropolis of Kuala Lumpur, Malaysia.

## METHODS

A cross-sectional survey was conducted among residents of the Community Housing Projects of Lembah Pantai, which is part of metropolitan Kuala Lumpur, Malaysia. Data were collected using face-to-face interviews. The households were selected from four Community Housing Projects (PPRs): Kampung Limau, Sri Pantai, Sri Cempaka, and Pantai Ria, which are situated in the catchment area of the planned recreational park. The PPRs were developed by the Kuala Lumpur City Hall and only given to families whose income is below MYR 2,500, with at least one child and who does not own a property within Kuala Lumpur. The total population in Kuala Lumpur is 1.67 million. Kuala Lumpur is considered among the most densely populated states in Malaysia (6,891 persons/km^2^).^15^ Community Housing Projects of Lembah Pantai consists of 1,896 units, and the majority have household size of 3 to 6 (mean household size, 4.66).^16^

### Sample size calculation and sampling procedure

The sample size was calculated using two ways. First, the sample size was calculated using the OpenEpi programme: since there was no previous study of PA levels among the low-income population, we assumed that the percentage of people achieving recommended PA is 50% (+ or −5%), with 95% confidence interval and 80% power of the study, and we calculated a sample size of 384 individuals. Second, the sample size was calculated using the Power and Sample Size programme and was done by taking the difference between the means of HRQoL scores from both inactive and active samples.^17^ The mean difference in scores was 2.9, with a standard deviation (SD) of 10.073. In order to achieve a power of 80% and significance level at 5%, the minimum sample size required in each group was 190. Thus, a total of 380 samples were needed to run the study. For both calculations, we inflated the sample size to be around 760 in order to account for a low respond rate.

Proportionate simple random sampling was used to obtain the number of participants from the four PPRs. Participants for this study were selected randomly from the PPRs, which required participants to be 18 years old and above and not confined in movements. Data were collected between May 1, 2015 and June 16, 2015. Altogether, 680 responded to our survey, which is an 88.5% respond rate. Of the 680 respondents, about 80.2% had complete data for PA, and 99.9% had complete data for HRQoL. Extreme values and incongruent values were subsequently excluded from the analysis.

### Measurements

#### Socio-demographic and socio-economic characteristics and presence of chronic disease

Socio-demographic data collected from participants included age, gender, ethnicity, family type, and marital status. Socio-economic data collected included education level, household income, current occupation, and most frequent means of commuting. Presence of chronic disease was also elucidated and its data collected accordingly.

#### Physical activity

The Global Physical Activity Questionnaire (GPAQ) short form version 2.0. was used to measure PA among the respondents. It was developed by the WHO to measure population PA.^18^

The GPAQ version 2.0 has been standardized to be reliable and valid in different settings (eg, culture and language). The GPAQ has been validated and used before in a study population whose commonly spoken language was Bahasa Malaysia.^18^^–^^20^ The GPAQ algorithm was used to classify weekly PA into two groups: individual who achieve recommended PA level (≥600 MET-minutes week^−1^) as “active” and individuals with PA <600 MET-minute week^−1^ as the “insufficient” group.^21^

#### Health-related quality of life

The SF-12v2 was administered to assess the HRQoL among the population under study. It is one of the most popular measurements of HRQoL and consists of physical and mental health scores, which are measured under various dichotomous, ordinal, and frequency scales. Eight scales are usually the result of the scores generated: Physical Functioning (PF), Role-Physical (RP), Bodily-Pain (BP), General Health (GH), Vitality (VT), Social Functioning (SF), Role-Emotional (RE), and Mental Health (MH). These scales are further summarized into comparable estimates of Physical Component Summary (PCS) and Mental Component Summary (MCS).

SF-12v2 has been validated in many countries among healthy and unhealthy populations; it has also been translated into many languages and validated across the globe, including in Asian populations.^22^^–^^30^ The SF-12v2 was used because it was less time consuming to fill questionnaire and has comparable accuracy to the longer SF-36.^31^ The scores range from 0 to 100: scores of 100 signify optimal health, while scores that are more or less than 50 are more or less healthy than the American population mean. Thus, a higher score indicates a higher QoL. Low scores on the bodily pain scale are typical of a person who has very severe and extremely limiting pain, and high scores represent individuals who have no pain or pain-related limitations.

### Statistical analysis

Descriptive analysis are presented as means and SDs for quantitative variables or as a proportion for categorical variable using SPSS version 20 (IBM Corp, Armonk, NY, USA).^32^ Normality was checked using the Shapiro-Wilk Test.

Age was categorized into five categories (18–29, 30–39, 40–49, 50–59, and ≥60 years); ethnicity (Malay, Indians, Chinese, and others), family type (nuclear, extended, and non-related house members [NRHM]), marital status (single, married, divorcee, and widow/widower), education (no formal education, primary, secondary, post-secondary, university and others), household income (<RM 2,000, RM 2,000–2,999, RM 3,000–3,999, and ≥RM 4,000), current occupation (paid employed, self-employed, retire, housewife, unemployed and others, which include: those studying, studying and working and those that don’t know or can’t specify the category which they belong to), most frequent means of commuting (bus, car, motorcycle, walking, and other), and presence of chronic diseases.

The relationships between PA and respondents’ characteristics (demographic, socio-economic, and presence of chronic diseases) were assessed. PA was the outcome (dependent variables), while the respondents’ characteristics were the predictor (independent variables). This relationship was assessed using one-way analysis of variance and independent *t*-test, with appropriate post-hoc analysis done for multiple comparisons. Also, prevalence/proportion of physical inactivity was assessed with the use of chi-square. The levels of PA (active/inactive) were the outcome, while the respondents’ characteristics were the predictors. Analysis of co-variance was carried out to find the actual interaction between the independent variable (PA) and the dependent variable (domains of QoL), with adjustment for age as a continuous variable. Multiple linear regression was carried out to find the actual interaction between the independent variable (PA) and the dependent variable (commuting mode) with adjusting for confounding variables as age, gender, ethnicity, presence of chronic illness, marital status and household income.

Another multiple linear regression was carried out to find the actual interaction between the independent variable (PA) and the dependent variable (domains of QoL), with adjustment for age, gender, ethnicity, presence of chronic illness, marital status, and household income as confounding variables.

### Ethics approval

The design of this study followed international guidelines. It was subsequently approved by the Ethics Committee of the University Malaya Medical Centre (Ref. no. 944.18). Participants voluntarily participated in the study, and written informed consent was obtained from all participants.

## RESULTS

### Demographic and socio-economic characteristics, presence of chronic disease, and health-related quality of life

A total of 667 participants of at least 18 year old (mean age 44.4 years [SD, 14.9]) responded to the questionnaires, the numbers are distributed more or less equally among the age groups. There is almost an equal distribution among the gender (51.1% males and 48.9% females); Malays (83.3%), members of Nuclear families (79.2%), married (74.1%), with at least secondary school education (63.7%), employed (57.6%), low-income earners of <RM 2,000 (37.4%), and many walk as a means of commuting (51.1%) (Table 1). The majority were healthy respondents (66.8%), with 33.2% of the study participants having one or more chronic diseases. It was also observed that self-reported QoL among respondents was fair, with a mean PCS of 51.1 (95% CI, 49.5–50.8) and a mean MCS of 52.8 (95% CI, 52.1–53.4).

**Table 1.** Variation of physical activity and the prevalence of physical inactivity level by socio-economic factors

Variables	Total PA MET-min/week			Prevalence of physical inactivity (<600 MET-min/week)		
	
	*N*	Mean (95% CI)	*P*-value	*N*	%	*P*-value
**Age, years (*n* = 546)**						
18–29	107	5,132.1 (4,402.2–5,862.0)		96	4.7	0.000
30–39	96	4,032.8 (3,307.2–4,758.5)	0.000^a^		15.6	
40–49	125	3,496.0 (2,894.6–4,097.4)			16	
50–59	112	3,658.5 (2,993.3–4,323.7)			23.2	
≥60	106	3,004.7 (2,394.3–3,615.2)			28.3	

**Gender (*n* = 546)**						
Male	274	3,880.9 (3,435.4–4,326.3)	0.035	96	17.9	0.911
Female	272	4,659.9 (3,414.0–4,219.7)			17.3	

**Ethnicity (*n* = 546)**						
Malay	455	3,725.4 (3,399.9–4,050.9)	0.004^a^	96	18.2	0.474
Chinese	16	1,967.5 (973.4–2,961.5)			25.0	
Indians	72	5,039.7 (4,147.3–5,932.1)			12.5	
Others	3	4,053.3 (677.6–8,783.7)			0.0	

**Family type (*n* = 626)**						
Nuclear	496	4,109.0 (3,752.1–4,466.0)	0.020^a^	92	15.2	0.039
Extended	118	3,178.1 (2,561.6–3,794.6)			24.6	
NRHM^b^	12	2,583.3 (586.8–4,579.7)			25.0	

**Marital status (*n* = 545)**						
Single	112	4,659.1 (3,900.0–5,418.1)	0.062	95	12.5	0.356
Married	404	3,631.4 (3,296.2–3,966.7)			18.8	
Divorcee	21	3,828.5 (2,536.1–5,121.0)			14.3	
Widow/Widower	8	4,015.0 (1,025.6–7,004.3)			25.0	

**Education (*n* = 546)**						
No formal	11	3,618.1 (1,615.5–5,620.7)	0.408	96	9.1	0.307
Primary	96	3,322.7 (2,693.2–3,952.1)			26.0	
Secondary	348	4,104.0 (3,713.2–4,494.9)			16.1	
Post-secondary	61	3,500.1 (2,552.2–4,448.0)			14.8	
University	24	3,322.5 (1,991.4–4,653.5)			16.7	
Others	6	3,553.3 (1,045.0–6,061.7)			16.7	

**Household income (RM) (*n* = 523)**						
<2,000	196	3,416.1 (2,962.3–3,869.9)	0.120	91	21.9	0.114
2,000–2,999	173	4,075.3 (3,524.8–4,625.9)			13.3	
3,000–3,999	88	4,153.3 (3,353.8–4,952.7)			13.6	
≥4,000	66	4,433.2 (3,435.2–5,411.3)			19.7	

**Current occupation (*n* = 545)**						
Paid employed	215	3,695.3 (3,209.6–4,181.0)	0.011^a^	96	15.8	
Self-employment	99	4,831.9 (3,989.7–5,674.1)			18.2	0.062
Retire	40	3,770.5 (2,582.3–4,958.6)			27.5	
Housewife	119	3,734.1 (3,195.7–4,272.4)			16.0	
Unemployment	46	2,559.1 (1,737.2–3,380.9)			28.3	
Others	26	4,424.0 (3,125.8–5,723.1)			3.8	

**Commuting (*n* = 536)**						
Bus	34	4,398.1 (3,248.8–5,547.3)	0.000^a^	95	8.8	0.130
Car	82	4,018.3 (3,189.0–4,847.5)			15.9	
Motorcycle	129	4,995.2 (4,306.7–5,683.6)			13.2	
Walking	274	3,176.4 (2,794.4–3,558.4)			21.9	
Others	17	5,642.8 (4,039.0–7,246.5)			11.8	

**Presence of chronic disease(s) (*n* = 546)**						
None	365	4,022.0 (3,647.3–4,396.6)	0.121	96	14.5	0.015
1–2	163	3,612.6 (3,076.4–4,148.7)			22.7	
3 and more	18	2,482.2 (1,313.7–3,650.7)			33.3	

^a^Post hoc showed significant difference.

^b^NRHM, Non-related household members (eg maid, worker).

NB: The association was analysed by ANOVA and independent *T*-test; with Post-Hoc test done where necessary. Chi-square and Fisher exact tests were used for the association between socio-demographic factors and physical inactivity. Significant levels at *P* < 0.05.

### Physical activity

The level of PA is presented in Table 1, and its relationships with respondents’ characteristics are outlined. Table 1 also shows that the PA differs significantly between age groups, where PA reduces with increasing age in the total PA. Females were significantly more physically active than males. Indians had significantly higher total PA (*P* = 0.004) than the other ethnic groups. Members of nuclear families had a higher level of total PA (*P* = 0.020). As expected, singles had the highest level of PA, but the result was not significant (*P* = 0.062). In term of occupation, self-employed had the highest total PA (*P* = 0.011). Across the income groups, there was no significant difference observed. Across means of commonest commuting, others group followed by motorcyclists group reported significantly higher total PA (Table 1 and Table 2).

**Table 2.** The pattern of physical activity domains by commuting mode

Variable	MVPA at work (MET-min day)			MVPA at transport (MET-min day)			MVPA at sport (MET-min day)		
		
	Mean	SE	*P*-value	Mean	SE	*P*-value	Mean	SE	*P*-value
**Commuting (*n* = 536)**									
Bus	580.2	126.4	0.642	76.4	13.6	0.146	58.9	63.6	0.023^a^
Car	643.6	129.8		33.2	13.6		275.2	66.2	
Motorcycle	484.4	129.6		30.8	13.7		82.0	66.3	
Walking	415.8	86.4		65.1	8.9		77.6	44.6	
Others	431.7	153.4		41.5	16.1		27.9	84.0	

MET, metabolic equivalent; MVPA, moderate-to-vigorous physical activity; SE, standard error.

^a^Significant at *P* < 0.05.

The model Adjusted for age, gender, ethnic group, marital status, household income and presence of chronic illness.

Overall, 17.6% (95% CI, 14.3–20.9%) of the respondents did not achieve the recommended WHO PA (≥600 MET-minutes week^−1^). It should be noted that physical inactivity significantly increased with increasing age (*P* ≤ 0.001), among non-related household members (*P* = 0.039), with increasing number of chronic disease(s) (*P* = 0.015), and among unemployed and retired individuals (*P* < 0.1) (Table 1).

Table 3 shows that 29.8% of the study population reported no active-commuting (Mean MVPA_tranport_ MET-minutes/day 50.4; SD, 63.2), 24.3% reported no active occupation (mean MVPA_work_ MET-minutes/day 423.2; SD, 583.3), 57% reported no active recreational activity (mean MVPA_sport_ MET-minutes/day 39.9; SD, 69.6), and 32.9% reported sedentary behavior of 4 hours or more (mean MET-minutes/day 235.1; SD, 180.7).

**Table 3.** Descriptive data of physical activity domains

Physical activity domain	Mean	Standard deviation
MVPA_sports_ METmin/day	39.9	69.6
MVPA_work_ METmin/day	423.2	583.3
MVPA_tranport_ METmin/day	50.4	63.2
Sedentary-METmin/day	235.1	180.7

MET, metabolic equivalent; MVPA, moderate-to-vigorous physical activity.

### Physical activity and quality of life

The relationship between levels of PA and the domains of SF 12v2 QoL was assessed using one-way analysis of co-variance with adjustment for age and using multiple linear regressions with adjustment for age, gender, ethnicity, presence of chronic illness, marital status and household income.

Regarding the 10-domain scales scores, between-PA group differences were investigated. Across domains, the active group had a trend of higher level of QoL. After inferential analysis, the active group had significantly higher BP (*P* < 0.05), MH (*P* < 0.05), and MCS (*P* < 0.1) scores than the insufficient group (Table 4 and Table 5).

**Table 4.** Adjusted HRQOL measures in respondents among physical activity groups

Quality of life domain	Insufficient mean (SD)	Sufficient mean (SD)	*P*-value	F^c^
Physical Composite Score	49.33 (8.50)	50.17 (8.48)	0.633	0.229
Physical functioning	77.34 (29.26)	77.04 (29.60)	0.711	0.138
Role physical	80.33 (24.31)	81.85 (23.97)	0.505	0.444
Bodily pain^d^	71.35 (30.77)	79.54 (27.58)	0.018^a^	5.650
General health	58.49 (27.59)	64.42 (27.24)	0.411	0.677
Mental Composite Score	50.93 (9.00)	53.17 (8.47)	0.070^b^	3.303
Vitality	69.53 (26.45)	74.43 (24.82)	0.416	0.662
Social functioning	81.77 (25.77)	84.26 (23.58)	0.442	0.592
Role emotional	76.43 (23.91)	79.50 (24.23)	0.280	1.168
Mental health	71.48 (21.12)	77.60 (19.78)	0.032^a^	4.608

HRQOL, health-related quality of life; SD, standard deviation.

^a^Significant at *P* < 0.05.

^b^Significant at *P* < 0.1.

^c^ANCOVA adjusted for age.

^d^Low scores on the bodily pain scale are typical of a person who has very severe and extremely limiting pain, and high scores represent individuals who have no pain or pain-related limitations.

**Table 5.** Linear regression model of adjusted HRQOL measures in respondents among physical activity groups

Quality of life domain	Unstandardized coefficient	Standardized coefficient^c^	*P*-value
Physical Composite Score	0.727	0.032	0.473
Physical functioning	−1.130	0.014	0.749
Role physical	2.032	0.032	0.479
Bodily pain	9.241	0.124	0.006^a^
General health	3.696	0.052	0.246
Mental Composite Score	1.864	0.082	0.068^b^
Vitality	2.649	0.040	0.369
Social functioning	1.700	0.027	0.547
Role emotional	3.688	0.057	0.202
Mental health	5.122	0.096	0.031^a^

HRQOL, health-related quality of life.

^a^Significant at *P* < 0.05.

^b^Significant at *P* < 0.1.

^c^Adjusted for age, gender, ethnic group, marital status, household income and presence of chronic illness.

## DISCUSSION

The current research was designed to study the prevalence of PA in relation to different socio-economic statuses and the association between physical inactivity with HRQoL in low-income Malaysian adults. Highest PA was reported among those of 18–29 years old; at same time, physical inactivity increased with age in this study. These results are consistent with those reported in NHMS-2015 and in all WHO regions, a pattern recognized to have a strong biological basis.^33^

Higher PA was also reported in females compared to males, among the Indian ethnic group, and among those from a nuclear family. Generally, this study shows a low prevalence of physical inactivity among the Malaysian population (17.6%), which is similar to that reported in South East Asia (17.0%) and lower than that estimated globally (31.1%), in the Western Pacific Region (33.7%), in the Americas (43.3%), in the Eastern Mediterranean (43.2%), and in Europe (34.8).^34^ A study published in 2017 showed that the average step counts of 3,787 Malaysian users was 3,963 steps/day, a PA level that is quite low compared to the global average. Although we suspect the data has serious bias, such as smartphone carrying time (depending on the types of clothing differed by country), the objective PA data give important insights into the understanding of physical inactivity prevalence.^35^ Generally, there is a clear worldwide variation in physical inactivity prevalence, where physical inactivity is more common in countries of high income than in those of low income. However, globally and regardless of income level of a country, the low-income people had more physical inactivity than those with high income.^34^

Education and income levels are considered as proxies of socio-economic status. However, this study shows no significant association between those factors and the prevalence of PA and inactivity. Previous studies in Chile reported higher levels of physical inactivity among the low-socioeconomic-status group,^33^ in contrast to that reported in Mexico^36^ and Brazil,^37^ where higher physical inactivity was found among the high-socioeconomic-status group. These variations could be due to falling occupational PA, which is high among low-socioeconomic-status groups, and increasing leisure PA, which mostly occurs among the high-socioeconomic-status groups. Since our study is conducted in an area with a large low-income population, we were unable to find differences among socio-economic status. Self-employed workers are reported to have the highest PA, which is consistent with data reported in NHMS-2015 but contrasts with that reported in a study done in Nepal.^38^

In the present study, there was a significant relationship between physical inactivity and having chronic illness. In 2009, physical inactivity was recognize as the 4^th^ foremost risk factor for NCDs and accounted for more than three million preventable deaths.^34^ To prevent such deaths, policy makers need to know about the PA level in the population for implementation of effective NCDs prevention programmes. In addition, these data could help in the development of some strategies and polices to increase the level of PA, especially for active commuting and reducing the NCD burden. However, our cross-sectional study design does not permit identification of a causal association between PA and chronic illness.

Active commuting (eg, walking or cycling) has essential benefits to reduce the risk of mortality from NCDs.^39^ Our study estimates that 51.1% of the population had an active commute. This percentage in Malaysia is higher than that reported in other literature from Australia, Canada, the United States, the Netherlands, and China, with respective active-commuting rates of 4.7%, 7.8%, 10.4%, 37.9%, and 46.1%,^34^ and lower than Chile (66%).^33^

Another PA-related domain that has been extensively studied over the last decade is sedentary-related behavior.^40^ Increasing sedentary behavior is strongly related to an increased risk of NCDs.^41^ In this study, this prevalence is lower (32.9%) than that found worldwide (41.5%)^34^ and for Argentina (52.8%), but it is more than that found for Brazil (28.2%) and Colombia (27.2%).

The findings of our study showed that the recommended level of PA is positively associated with some domains in physical and mental aspects of HRQoL. Meeting the recommended level of PA was significantly associated with better scores on BP, MH, and MCS after the adjustment of confounders. A study done in Malaysia found that the prevalence of chronic pain among elderly individuals was 15.2% (95% CI, 14.5–16.8%) and that there is an increased prevalence with advancing age.^42^ Simultaneously, in Malaysia, there is an increasing trend of mental health problems among adults, from 10.7% in 1996 to 29.2% in 2015.^13^ The prevalence is higher among adults from low-income families and younger age group.^13^ For that, health benefits from regular exercise should be highlighted and reinforced by health care providers, particularly mental health professionals. PA interventions for persons suffering from chronic pain or serious mental illness could provide effective, evidence-based findings in order to understand the influence of combining such interventions with traditional health management.

This research is the first to study the association between the recommended levels of PA and HRQoL in Malaysia. Previous studies in elsewhere also found that the WHO recommended level of PA was also positively associated with some domains of HRQoL. In France, researchers found that participants who attained the WHO recommended PA level had higher scores in almost all domains of QoL than those did not attain the recommended PA level.^17^ In Croatia, researchers found significant associations of PA with vitality, MH, and MCS in females and with PF, BP, SF, MH, and PCS in male participants.^43^

Many studies have reported a positive association between PA and HRQoL (and its domains). Many studies have 60–100% agreement of results using the 36-item and the 12-item short form health surveys developed for the Medical Outcome Study (MOS SF-36 and SF-12, respectively) and the WHO Quality of Life shorter version (WHOQoL-BREF); though with some inconclusive associations and lack of association occasionally.^44^ The direction of the association across studies showed increased level of HRQoL with higher PA among healthy, unhealthy, and elderly individuals.^45^^,^^46^

The variations in the findings of our study and previous studies were due to some possible reasons. First, the previously mentioned studies used either one specific PA domain or four domains of PA as independent variables (work, transport, domestic, and leisure); however, we used two categories (active and insufficient). Second, the previous studies reported the results of subgroup analysis according to gender.

### Strengths

Using of GPAQ, which is used worldwide, to study the PA pattern in this research could enable the comparison of the result with other countries globally. We collected the reported PA and HRQoL in the catchment area of a planned recreational park so that we can follow up and evaluate the effect of infrastructural development in PA and HRQoL among the residents of the catchment area. In addition, the high response rate (88.5%) is an another strength of this study.

### Limitations

Self-reporting is unreliable because the housework and occupational PA, especially in low- and middle-income participants, are often not considered part of PA, as such activities related to housework, occupation, and transport are mixed with other activities of daily life. Because of the study design we used, we are unable to assess the causal association between PA and chronic illness. In addition, the survey is only a questionnaire, we did not measure BMI, and the questionnaire did not include an assessment of smoking status.

### Conclusion and recommendation

In summary, our result shows that almost one out of five low-income adults were physically inactive, and the level of PA and inactivity was significantly associated with some socio-demographic factors, particularly with age and presence of chronic illness. It is essential to develop culturally sensitive public health interventions which can also accommodate people with chronic illness. In addition, we identified that individuals attaining recommended levels of PA had better scores on some domains of HRQoL, particularly for mental health. It is a promising possibility to use PA intervention to reduce the mental health problem in the general population.
